# Risk factors to mental health challenges among the LGBTI+ community in Gaborone, Botswana

**DOI:** 10.4102/curationis.v47i1.2543

**Published:** 2024-05-22

**Authors:** David S. Mangwegape, Mofatiki E. Manyedi, Boitumelo J. Molato

**Affiliations:** 1 Department of Psychiatric Mental Health Nursing, Institute of Health Sciences, Lobatse, Botswana; 2 School of Nursing, Faculty of Health Sciences, North-West University, Mahikeng, South Africa

**Keywords:** coping mechanisms, discrimination, hormonal replacement therapy, LGBTI+, mental health challenges, misgendering, stigma

## Abstract

**Background:**

Lesbian, Gay, Bisexual, Transgender, Intersex and other gender diverse groupings symbolised by + (LGBTI+) individuals experience adverse mental health problems, and several factors have been documented to facilitate such problems. However, in Botswana, the factors facilitating LGBTI+ individuals to experience mental health challenges have not been explored with previous studies only highlighting the poor mental health outcomes they experience.

**Objectives:**

The aim of the study was to explore and describe factors that could cause mental health challenges in LGBTI+ individuals in Gaborone, Botswana.

**Method:**

A qualitative, descriptive, phenomenological design was employed to examine the research question. In data collection, 15 unstructured in-depth telephonic interviews were conducted until data saturation. Data were analysed with a co-coder using the data analysis method by Colaizzi.

**Results:**

Three themes emerged following data analysis and were reasons for experiencing mental health challenges, experiences of challenges in accessing healthcare services and the social challenges of everyday life.

**Conclusion:**

The findings indicate that a variety of factors influence the mental health problems in some LGBTI+ individuals.

**Contribution:**

The knowledge of the factors that cause LGBTI+ individuals’ mental health challenges can inform mental healthcare to be rendered. The findings can apprise nursing curriculum development and policy regarding the needs of LGBTI+ individuals.

## Introduction

Evidence is abounding that individuals classified under sexual minorities experience health disparities when compared to heterosexual and cisgender populations (Wittgens et al. 2022:357). Sexual minorities encompass individuals identifying as Lesbian, Gay, Bisexual, Transgender, Intersex and other gender-diverse sub-groupings symbolised by the acronym LGBTI+. The authors acknowledge that there are several other acronyms, but the article will consistently use LGBTI+ to refer to participants in the study while other acronyms will be used as in the studies they were cited in.

Across the globe, LGBTI+ individuals are marginalised, harassed, victimised and also endure discrimination at individual, institutional and structural levels (Meer et al. 2017:6). However, efforts have been made to deal with stigma and discrimination. As an example, several countries have had same-sex marriages legalised, notably some states in United States (US), Ireland and South Africa (Luvuno et al. 2019:1). Additionally, some countries have passed anti-discrimination laws that help to stop stigma and discrimination (Mendos et al. 2020:26).

Despite the above laudable developments, there is documented evidence that LGBTI+ individuals are increasingly facing mental health challenges (Mangwegape et al. 2023). LGBTI+ individuals face several health disparities and have increased risk for mental health problems (Luvuno et al. 2019:1). A study conducted US highlighted that ‘post-traumatic stress disorder, depression, anxiety, binge eating, problematic anger, multiple somatic symptoms, and insomnia’ were mental health problems likely to be experienced by LGBTI+ individuals in comparison with heterosexual individuals (Carey et al. 2022:521; Hafeez et al. 2017:1).

Understanding the causes of mental health disparities among LGBTI+ individuals is very important (Mongelli et al. 2019:47: Meer et al. 2017:5). There are varied reasons linked with the proliferation of mental health challenges among LGBTI+ individuals, but key among those are stigma and discrimination. Several studies and reviews have highlighted that stigma and discrimination expedite physical and mental health problems among LGBTI+ individuals (Cyrus 2017:194; Hunt et al. 2017:2; Valente, Dworkin & Bockting 2022:1445). A review by Zeeman et al. (2019:974) asserts that LGBTI+ individuals experience the inequalities brought by heterosexism, minority stress, experiences of stigma and victimisation.

Mental health of LGBTI+ individuals is also affected by issues such as difficulty to come out, a lack of acceptance and isolation (Ventriglio et al. 2022:200). Social unacceptance often facilitates issues that are significantly detrimental to the wellbeing of LGBTI+, a situation often facilitated by religious establishments (Sandfort & Keddy, cited in Meer et al. 2017:41).

A study conducted in Botswana found that persons who identified as gays, lesbians and bisexuals (GLB) experienced mental health challenges (Ehlers, Zuyderduin & Oosthuizen 2001:852). However, the study did not explore the underlying factors that predispose them to psychiatric problems and also focused only on three sexual minority groupings. Moreover, a review of the aforementioned study recommended that studies be conducted to determine predisposing factors to mental health challenges among the LGBTI+ community (Selemogwe & White 2013:409).

Suen et al. (2020:2302) have decried limited research on individuals in the sexual and minority population. Furthermore, the distinctive challenges and the needs of this sexual minority group are misunderstood by many people (Klein 2017:207). In light of this assertion, only one study to the authors’ knowledge has been conducted regarding mental health challenges among the LGBTI+ individuals in Botswana. There is growing evidence that several factors facilitate mental health challenges among sexual minorities, but there is dearth of literature on the phenomenon in Botswana and thus the need to explore and describe factors that facilitate mental health problems among LGBTI+ individuals in Gaborone, Botswana. The findings of the study could be utilised to help guide nursing care and inform curriculum development as far as mental healthcare of LGBTI+ individuals is concerned.

## Objective

The research was aimed at exploring and describing factors that facilitate mental health challenges in LGBTI+ individuals in Gaborone, Botswana.

## Research methods and design

### Study design

This article is part of a broader study that employed a qualitative, descriptive, phenomenological design. Phenomenology is a research design that explores a phenomenon from the perspective of those that experience it (Teherani et al. 2015:670). The design was suitable as the phenomenon under study was on the lived experiences of LGBTI+ individuals, precisely exploring the factors that lead to their mental health challenges (Morrow, Rodriguez & King 2015).

### Setting

According to Statistics Botswana (2022:5), Gaborone has an approximate population of 289 703 people. Gaborone, which is the capital city of Botswana, was the ideal research setting because there were diverse individuals from which study participants could be identified from.

### Study population

The inclusion criteria for the study related to individuals who self-identified with one of the LGBTI+ groupings, resided in Gaborone and were at least 18 years of age.

Fifteen participants made the sample of the study. Data saturation was reached at the 12th participant while three more participants were involved in the interviews for confirmation of the findings.

### Sampling strategy

Network sampling takes advantage of social networks; hence participants were established through the advocacy organisation LEGABIBO (Lesbians, Gays and Bisexuals of Botswana) who were the gatekeepers (Grove 2023:268–269). This sampling method is often used with minority groups or communities that are not easily accessible. The first participant was identified through a mediator and each participant was involved in identifying other subsequent participants.

### Data collection

Data were collected from June 2021 to August 2021 through unstructured and in-depth individualised interviews with an interview guide. The interview guide was developed in English and Setswana. Individual interviews were conducted in either English or Setswana, as per participant preferences. Data that were collected telephonically to minimise coronavirus disease 2019 (COVID-19) transmission were captured by the use of a voice recorder. Each participant was anonymised through the use of numerical codes from the first participant denoted as P1 to the last participant indicated as P15. In ensuring that data were protected, they were stored in a computer encrypted by a password. Data from interviews conducted in Setswana were then translated. The first author then transcribed the recorded interviews, which were from 45 min to 65 min long before analysis.

### Data analysis

Data were analysed manually using Colaizzi’s data analysis method (Polit & Beck 2022:263). A co-coder was engaged to code the themes, which were mutually agreed upon, with input from the second author. As suggested by Morrow et al. (2015), the analysis underwent a seven-step process that encompasses the following:

- Familiarisation of the data by researchers.
- Identifying statements that are significant to the phenomenon being investigated.
- Formulating meanings from the significant statements.
- Clustering meanings into common themes.
- Writing an exhaustive description of the phenomenon.
- Condensing the exhaustive description into short fundamental structure.
- Verifying the fundamental structure as to whether it captures participants’ experiences by asking some participants themselves.

### Trustworthiness

Trustworthiness was ensured through use of Lincoln and Guba’s framework (Polit & Beck 2017:560). Strategies like credibility, transferability, dependability, confirmability and transparency were utilised to ensure trustworthiness of the study. In ensuring transparency, there was prolonged interview with a significant number of participants. After data saturation that occurred at 12 participants, three more participants were interviewed to ensure that rich data were collected. The interviews were audio-recorded and then transcribed verbatim. An independent co-coder was engaged to ensure the integrity of the findings. The operational framework of the research process was availed for the study to be done in a different context.

To ensure confirmability, the fields notes of the interviews and transcriptions in verbatim format were availed. Authenticity of the findings was ensured by providing a thick description with rich quotes in the description of each theme. The first author wrote his personal reflections in a reflective journal during data collection and analysis, an undertaking that enhanced the credibility of the study (Polit & Beck 2022:279). In order to put the researchers’ assumptions and ideas on the plight of LGBTI+ individuals in abeyance, bracketing was carried out by the researcher (Shorey & Eng 2022:1969).

### Ethical considerations

The study was approved by the Ministry of Health and Wellness (Ref: HPDME 13/18/1). Goodwill permission was given by an LGBTI+ advocacy organisation.

Participants voluntarily took part in the study and were advised of their right to withdraw at any time. Both verbal and written informed consent were solicited from the participants.

## Results

The data of the 15 participants were obtained from them during the interviews. The participants had different gender identities as summarised in Table 1.

**TABLE 1:** Socio-demographic profile of the participants.

Participant	Gender
1	Gender non-conforming
2	Gender non-conforming
3	Gay
4	Transgender woman
5	Bisexual
6	Lesbian
7	Transgender man
8	Transgender woman
9	Transgender man
10	Transgender man
11	Transgender woman
12	Intersex
13	Intersex
14	Intersex
15	Lesbian

Three themes emerged as findings of the study, being the reasons for experiencing mental health challenges, experiences of challenges in accessing healthcare and social challenges of everyday life. The themes, sub-themes and sub-subthemes are summarised in Table 2.

**TABLE 2:** Themes and sub-themes.

Themes	Sub-themes	Sub-subtheme
1. Reasons for experiencing mental health challenges	1.1 Experiences of stigma and discrimination	1.1.1 Lack of support and having to cope with the situation alone 1.1.2 Experiences of rejection 1.1.3 Difficulty in keeping and obtaining employment 1.1.4 Experiences of unhealthy family relationship
	1.2 Experiences of being misgendered	-
	1.3 Unhealthy coping mechanism	-
2. Experiences of challenges in accessing healthcare services	2.1 Avoiding healthcare due to stigma	2.1.1 Experience of insecurity to seek counselling 2.1.2 Experiences of having to undress unnecessarily or being asked inappropriate questions
3. Social challenges of everyday life	3.1 Problems related to issues of identity	3.1.1 Difficulty in affording expenses related to hormone treatments and surgeries 3.1.2 Frustration in managing romantic, intimate relationships 3.1.3 Having to deal with people not familiar with transgender identities

*Source*: Adapted from Mangwegape, D.S.M., 2022, ‘Mental health challenges among the lesbian, gay, bisexual, transgender and intersex community in Gaborone, Botswana’, Masters dissertation, North-West University, p. 36

### Theme 1: Reasons for experiencing mental health challenges

The findings revealed that there were various factors that led to LGBTI+ individuals experiencing mental health problems. They include being subjected to stigma and discrimination, experiences of being misgendered and unhealthy coping mechanisms.

#### Sub-theme 1.1: Experiences of stigma and discrimination

Stigma and discrimination emerged as one of the reasons that facilitate mental health challenges for the LGBTI+ individuals. The participants experienced stigma and discrimination through various forms being verbal, non-verbal, physical and sexual. Some LGBTI+ individuals in the context of the study were addressed by disparaging and discriminatory words like ‘bastards’ and sinners and innuendos such as ‘those that stop rain’, and *dibatana* [creatures] were used to shame them.

One of the participants expressed the following sentiment regarding discrimination:

‘Discrimination is one of the things that affect our mental health in the LGBTI+ community, all of it! A person would not want to share a seat in a taxi as when you board it, they disembark.’ (P1, Gender non-conforming)

Another participant echoed similar sentiments of discrimination and shared as below:

‘On social media, I saw a nurse who works in a local psychiatric hospital, but I can’t mention his name; he suggested that gays be infected by corona to die.’ (P2, gender-non-conforming)

Participants also narrated that discrimination is evident in public transport services for LGBTI+ individuals. They said that the discrimination led to them being abused; hence they end up suffering emotionally. The quote below attests to that:

‘It happens in public transport; we are suffering people as people are abusing us.’ (P3, gay)

However, some participants have shown that they spend time with their loved ones to avoid their mental health problems being triggered by discriminating posts on social media. This is what one participant said:

‘My mental health can also be influenced by what we see on social media. What I try to do is spend time with my boyfriend and stay away from my phone.’ (P4, transgender woman)

**Sub-subtheme 1.1.1: Lack of support and having to cope with the situation alone:** Frustrations on the lack of parental support, friends and sabotage from the family were factors that led to poor mental health among participants. Consequently, because of the lack of social support, they end up not seeking help. Below is what they said:

‘I can’t even ask for money from her, and I am craving for her support that I don’t get.’ (P2, gender non-conforming)

Study findings revealed that some participants dealt with challenges on their own as family and friends alike were not supportive. The participants lamented that they had to deal with adverse circumstances alone. The following quotes depict this:

‘You end up dealing with issues on your own.’ (P3, gay)

‘You cannot get the full support from the family; you cannot get full support from friends.’ (P6, lesbian)

Similarly, some participants revealed that in order to avoid mental health problems they have accepted that no one will support them. They said that they are used to a lack of support. One responded as follows:

‘I have never been supported and I have gotten used to it.’ (P7, transgender man)

**Sub subtheme 1.1.2: Experiences of rejection:** From the field notes, it was evident that the study participants felt rejected as they expressed that. Furthermore, some participants indicated that they felt worthless because of being rejected by the community members. The participants shared as below:

‘You don’t feel accepted, you don’t feel like you belong.’ (P1, gender non-conforming)

‘The major challenge is the lack of acceptance by the society and thus will need someone to help in nurturing self-worth.’ (P5, bisexual)

LGBTI+ individuals in the study also highlighted rejection at the family level. Rejection by family was more painful as they considered family to be the ideal first level of support. One participant highlighted:

‘We are not taken or accepted as ordinary people from family and friends or people we don’t know from the community and stuff …You find yourself having grown up with people then they get to realise that you are no longer that person, then they distance themselves from you.’ (P6, lesbian)

Participants also narrated experiences of being rejected when seeking help from service providers like the police department. This is depicted in the following statement:

‘When somebody identifying as LGBTI fought, there is drama at the police station. They would cause drama; they wouldn’t help you like they are helping a human being.’ (P1, gender non-conforming)

**Sub-subtheme 1.1.3: Difficulty in keeping and obtaining employment:** The participants had a challenge with keeping and obtaining employment because of stigma and discrimination. Additionally, some participants’ hindrance to employment prospects was the gender marker that was not aligned to the one on the identity document. They had this to say:

‘I will be told that I will influence kids to be LGBTI and I should do this, and I shouldn’t do this.’ (P1, gender non-confirming)

Some participants reiterated that they were not given opportunities because of their diverse gender identity. One participant who was shamed and not offered a modelling engagement said the following:

‘There is this other guy that I sent my profile to if he needs modelling services, I am available anytime, he told me that I am a devil, he does not want a curse in his business, does not want his things to get mixed up.’ (P2, transgender)

Finding employment was difficult when the identity card has not been changed to affirm the gender identity of some LGBTI+ individuals. Some participants narrated that as a means to survive, they resorted to sex work. One response was as follows:

‘The choices that I have made because there were even times when money was tight because I couldn’t get a job. I was applying and people see my ID and then lay low because they are confused … It was difficult to find a job, so sex work was something that I will find money through.’ (P11, transgender woman)

Some individuals identifying as intersex highlighted that their bodies are different and not in line with the binary options of male and female. As a result of this, they are often uncomfortable, which makes it difficult in finding employment. One participant, in disappointment, said the following:

‘Beard started growing when I long finished schooling. As a result of this I am unable to find employment.’ (P13, intersex)

**Sub-subtheme 1.1.4: Experiences of unhealthy family relationship:** Participants expressed their devastating and frustrating experiences of belonging to a family that is homophobic towards them. The participants expressed feeling disappointed as family members disowned them, kicked them out of their homes and neglected them. They said:

‘I have never had that good acceptance from family. My family have long rejected me when I was 18 years maybe let me say when I was growing.’ (P1, gender non-conforming)

‘Ooh; homophobia starts at family level from your parents not accepting that you are trans, they don’t accept you and are negative towards you. They neglect you.’ (P2, gender non-confirming)

Because the relationships with family members were so poor, some participants decided to do gender transitioning without informing them. The participant said:

‘It does not really mean that we have a relationship, we don’t talk. I don’t see them much; we grew up without constant communication with each other. When we see each other is just exchanging greetings and then that’s it. We have never talked about me transitioning or anything like that.’ (P7, transgender man)

In order to deal with some of the mental health challenges, some LGBTI+ individuals indicate that they often went for counselling. Families will often be brought on board as part of the therapy, but some participants were devastated that family members were not honouring appointments with counsellors. A disappointed participant shared:

‘There was a time I was called for counselling with both my family members, and they showed that they don’t care, they don’t wanna do anything with me.’ (P10, transgender man)

#### Sub-theme 1.2: Experiences of being misgendered

The participants expressed that they aspire to be addressed by proper pronouns and expressed being hurt and saddened by being misgendered. Participants voiced that their families, members of the community and health professionals were the ones that were often addressing them with improper pronouns. The following was depicted to substantiate this:

‘I indicated the pronoun to address me with so that you don’t get confused addressing me as a male, you understand? We have pronouns.’ (P2, gender non-conforming)

Some participants attributed being misgendered to their own mental health problems with one participant stating that:

‘When they get back home people are surprised you have breasts, you have changed the way you dressed and they start questioning that and that on its own can cause a mental breakdown because nothing hurts like being misgendered.’ (P4, transgender woman)

Furthermore, the issue of identity document that is not aligned to the gender one identifies with was a source of emotional distress for some participants. The participant said:

‘The other issue is that of names, there are names within the ID that I don’t want addressed to me. People will continue the name but since it’s in the ID you have nothing to say.’ (P9, transgender woman)

It is evident that some participants did not reveal their sexual orientation in order to avoid being misgendered. Highlighting the concealment of their gender identity, one participant said:

‘You are always cautious, you always trying not to let them misgender you in any way possible.’ (P10, transgender woman)

#### Sub-theme 1.3: Unhealthy coping mechanisms

The participants revealed that in order to cope with stressors as a result of stigma and discrimination, they utilised unhealthy coping mechanisms like using cigarettes, dagga and alcohol. On using alcohol as a coping strategy, participants said:

‘It’s like when I drink, I get to contain my habit, protect yourself, avoid those who make you sad, avoid those who does this to you. That’s it.’ (P1, gender non-conforming)

‘When I am drunk, I get happy. I get to see my problems and feel that I have overcome them.’ (P13, intersex)

The experience of stigma, marginalisation and not being offered support were the reasons mentioned by participants for their substance abuse. Dagga was one of the drugs that participants used to cope with stressors. This is what they said:

‘There is substance abuse. Substance abuse, drug abuse is common. I think mainly it’s because if you are marginalised or if people cast you aside it’s common that you use something to cope replacing social connections or networks with drugs … I have conditioned my mind that I can’t deal with my anxiety without weed (dagga). I have conditioned my mind that I can’t talk to people without taking weed.’ (P10, transgender man)

One participant also pointed out that they coped with stress through smoking:

‘Yeah, it helped a lot that time because of that stress; if you take a puff you forget about your troubles.’ (P12, intersex)

### Theme 2: Experiences of challenges of accessing healthcare

The second theme that emerged was that of experiences of challenges in accessing healthcare services. Emotions experienced by the participants were of hurt, anger, frustration regarding their access to healthcare. The theme had only one sub-theme of avoiding healthcare services and the sub-categories of experience of insecurity to seek counselling and experiences of having to undress unnecessarily or being asked inappropriate questions.

#### Sub-theme 2.1: Avoiding healthcare due to stigma

As per participants, despite having mental health challenges, the LGBTI+ individuals will rather not seek help because of stigma from healthcare workers. The participants revealed that they felt insecure when they had to go for counselling due to stigmatising tendencies from professional counsellors. Additionally, when seeking healthcare services, they were often required to undress even when not necessary. These experiences adversely affected their state of mental health.

**Sub-subtheme 2.1.1: Experiences of insecurity to seek counselling:** The participants expressed being uncomfortable and frustrated while seeking counselling and mental health services because most healthcare workers did not respect their confidentiality. This is how one participant mentioned about the lack of confidentiality and professionalism among the healthcare professionals:

‘I went to seek help for my mental health, and somebody sees me there and goes on to tell people that I am mad.’ (P1, gender non-confirming)

Some participants lamented that some healthcare professionals imposed their religious views on them. The participants did not therefore attend to counselling fearing that religious views may be imposed on them. One participant observed:

‘The next thing after this guy finishes with his counselling, he goes on to tell us about his church and that his prophet can deliver homosexuality so he is inviting us to his church so that we are delivered because we can’t be living this kind of lifestyle.’ (P3, gay)

Healthcare workers’ attitudes were also highlighted by some participants as leading them not to seek counselling services. Their sentiments are expressed as follows:

‘We will be in a relationship and having fights but fail to seek counselling services because we will not be received openly. We will not be treated in the same manner as those from heterosexual relationships.’ (P5, bisexual)

‘I am afraid that I might be exposing myself to people who are dangerous and that will increase my vulnerability by telling them that I am trans and they may use this to hurt me.’ (P11, transgender woman)

**Sub-theme 2.1.2: Experiences of having to undress unnecessarily or being asked inappropriate questions:** Having to undress for inappropriate reasons was one of the deterrents to LGBTI+ individuals seeking help. Participants expressed that they were made to undress for a group of nurses in order for them to look at how they looked. This is what one said:

‘Once they realize that it’s the first time to meet a certain condition, they will want to see. Even if you have brought headache, they undress you so that they see.’ (P13, intersex)

Participants also expressed disappointment at being asked inappropriate questions. They were infuriated by health workers ‘getting to see what it is like to be an individual identifying as LGBTI+’. This is how they responded:

‘When I access services like HIV/AIDS prevention and care, the nurses at the clinic and hospital in our city, are very rude and ask lots of unnecessary and very uncomfortable questions … They choose the path of mostly asking uncomfortable questions like what is between your legs, which at the time is completely unrelated or unnecessary because I want to be treated for headache and they will ask me what is in between your legs.’ (P11, transgender woman)

### Theme 3: Social challenges of everyday life

The participants expressed having social challenges that were detrimental to their mental health. The challenges included problems related to issues of identity, difficulty in affording expenses related to hormonal replacement therapy (HRT), frustration in managing romantic and intimate relationships and people not familiar with transgender identities.

#### Sub-theme 3.1: Problems related to issues of identity

The research participants expressed frustration at being questioned by police officers and being asked to strip off naked in order that verification is done to see whether they are the gender they purport to be. The participants were also overwhelmed by the contrasting gender markers in identity documents and those that they identified with.

Below are statements by participants regarding identity documents:

‘[*I*]t it can start raising eyebrows when I provide my identity card because. people become curious as to why is your identity card written male even though I see a woman standing in front of me.’ (P4, transgender woman)

‘I am a true man that has beard and everything, and everything unfortunately my identity documents indicate I am a female.’ (P7, transgender man)

‘Aah, others address me as a female, when they check the ID, they realise that I am not female.’ (P12, Intersex)

Participants expressed being uncomfortable when not being assisted in hospitals because of identity cards that do not conform to their physical appearance, and this is what one participant revealed:

‘I went to seek medical attention I found some woman who took my ID and insisted that she wants to see the owner of the ID; she went on to show other nurses and they were laughing, and they came to me. It was uncomfortable and I left without being assisted.’ (P8, transgender woman)

In addition, the difficulty of the process of changing the identity document as part of transitioning was deemed by participants as affecting their mental health. The following quote attests to that:

‘Like identity document change for instance ‘I have an ID and my ID says I am female. If you look at me, I will look different from what my ID says. Identity document change is one of the mental health challenges that we experience.’ (P10, transgender woman)

**Sub-subtheme 3.1.1: Difficulty in affording expenses related to hormone treatments and surgeries:** Participants attributed the lack of access to HRT as a source of mental health challenges. The participants endured some frustration due to the fact that they do not get HRT from government healthcare facilities, which forced them to get it in private facilities.

Some participants highlighted that the HRT that they seek from private healthcare facilities was very expensive and this was emotionally distressing for them. The statements below are what some participants said:

‘I speak for transgender women and trans men. They need hormonal replacement tablets, but they are expensive.’ (P2, gender non-confirming)

‘It really gives me stress. It is like we are not doing anything for ourselves, we are always thinking of hormonal therapy; there is nothing you can do for yourself, you can’t buy yourself anything.’ (P9, transgender man)

Issues related to non-compliance to prescribed HRT were raised by the participants. The failure to get HRT on time resulted in negative outcomes to mental health and physical well-being. One participant said:

‘Because currently in Botswana transgender people change through medicine, it is costly. Those things, you can go for three months without taking them because you are broke [*not having money*]. When you don’t take testosterone, it brings hormonal imbalance.’ (P10, transgender man)

On the contrary, some participants displayed bravery by coming up with ways that helped them to have access to HRT. In order to have money to buy HRT, they resorted to sex work. The following quote depicts what was said:

‘The thing is, when you can’t access HRT services in the country, I have realised that(sic) we resort to sex work. Sex work is criminalised, is unsafe, we are not able to get condoms and all of those, we engage in survival sex work which is unsafe and contract HIV to pay for HRT in private institutions … We use a lot of money because we are not getting it from the government … it is not affordable and sustainable.’ (P11, transgender woman)

**Sub-subtheme 3.1.2: Frustration in managing romantic intimate relationships:** Sadness, disappointment and frustration were expressed by the research participants regarding their lack of involvement in romantic relationships. They pointed out that they fear to get involved in romantic relationships because of fear of being judged by members of the community. This is what they said:

‘Somebody will get attracted to me and show love to me, the same society they are going to take him back from me. They will tell him unpleasant news. Telling him about being a faggot and this and that.’ (P1, gender non-conforming)

Some participants narrated challenges they endure in romantic relationships as only LGBTI+ individuals were interested in them. They found it difficult to have relationships with people from outside the LGBTI+ community. This was the response:

‘So it’s really difficult to find a partner who is not within the LGBTI; because some people are clueless or ignorant and only people know of gays and lesbian when you talk of trans they don’t know.’ (P10, transgender)

Some LGBTI+ individuals who were interviewed were not free in sexual intimacies with their partners. In order to avoid being judged by partners, they revealed that they had coitus in the dark in order to hide sexual organs from the partner. This is what was said:

‘When I am having sexual intercourse, I am not free; not knowing what I will tell my partner as to what that is.’ (P13, intersex)

In contrast, one participant shared experiences of having acceptance of heterosexual partners in romantic relationships. One individual who has not medically transitioned revealed having a partner who was still getting to know about her. The following was reported by the participant:

‘My boyfriend, this is his very first relationship with a trans gender woman, He is still learning a lot of things and he is still learning my comfortability with my genitals or my private parts.’ (P4, transgender woman)

**Sub-subtheme 3.1.3: Having to deal with people not familiar with transgender identities:** It was apparent from the study findings that community members were mostly not familiar with transgender identities. This finding provided an indicator of the more stigma meted on transgender individuals because of this lack of familiarity. The research participants expressed frustration and emotional distress when narrating how people view them. Their sentiments are depicted in the following quotes:

‘To make it worse is when you are in Botswana, most people won’t even know what trans people are, you see.’ (P1, gender non-confirming)

‘At first they thought I am just a gay person, so they were fine with gay because our hood was full of gays back then. It was fine, and I transitioned to be a woman and people started being nasty, discriminative and they started to have this hatred towards me.’ (P8, transgender woman)

‘They don’t know. They see me as a cisgender man.’ (P9, transgender man)

‘People were not aware of transgender people, and they were having nasty reactions towards trans people.’ (P11, transgender woman)

## Discussion

To the authors’ knowledge, this is the first study in the context of Botswana to explore factors that lead to mental health challenges among members of the LGBTI+ community. The findings have revealed several factors that facilitate mental health challenges among LGBTI+ individuals.

### Reasons for experiencing mental health challenges

Stigma and discrimination have been highlighted by the participants as one of the factors that compromise their mental health. A study by Lozano-Verduzco, Fernández-Niño and Baruch-Domínguez (2017:224) is consistent with the current findings as it established a linkage of discrimination with depressive symptoms and problematic substance use among the LGBTI+ community. Moreover, pervasive gender-based stigma significantly contributes to health disparities among mostly transgender individuals (Goldenberg et al. 2021:467; Valente et al. 2022:1445). Bayrakdar and King (2021:10) posit that a lack of support can be positively linked to incidents of discrimination, harassment and violence. This affirms that LGBTI+ individuals experience reduced psychopathological consequences when supported in their gender identity (Olson et al.^2016^:5).

LGBTI+ individuals face rejection from some members of the society, which triggers emotional distress (Jones & Hillier 2013:287). Rejection often manifests in elevated minority stress levels and negative mental health outcomes (Camp, Vitoratou & Rimes 2019:2353; Flores 2019:5). As in the study, family rejection increased participants’ misuse of substances (Watson & Veale 2018:115). Rejection can also be an issue in the workplace as LGBTI+ individuals, particularly transgender women, experience discrimination in accessing employment (Flores 2019:7; International Labour Organization [ILO] 2012:1–2; Valfort 2017:4). Some participants, as a result of transphobia, had difficulty in finding employment and had to survive through sex work, which is consistent with one study (Hughto, Reisner & Panchakis 2015:5). Gender transitioning also made it difficult on issues of employment-related matters as transgender individuals face adverse outcomes in the workplace because they are not treated as others (Davis & Yeung 2022:1).

Evidence is abounding that having a preferred gender marker as an LGBTI+ individual facilitates positive mental health (Restar et al. 2020:1). However, this was in contrast with the current study as the participants expressed having gender markers that they did not prefer.

Cooper et al. (2020:5) define misgendering as the act of labelling an individual with a gender that they do not identify with. Participants were misgendered leading to them being psychologically distressed, expressing feelings of worthlessness and not feeling authentic (Dolan et al. 2020:150; McLemore 2014:5, 2018:53). A study by Russell et al. (2018:3) found that proper use of pronouns and names for individuals identifying as LGBTI+ contributes to the reduction of depression and suicide.

Consistent with the current study, there is evidence that LGBTI+ individuals use alcohol, tobacco and dagga as a strategy to counter stressors (Adams, Asiasiga & Neville 2021:6; Buchting et al. 2016:4; Delahanty et al. 2019:164; Felner et al. 2020:118). In dealing with the stress and stigma in their lives, LGBTI+ individuals may resort to using drugs, alcohol or tobacco (Klein 2017:209). However, the findings of a study in Ireland demonstrate that identification as an LGBTI+ individual was a risk factor to mental health problems and not the problematic use of substances (Travers et al. 2021:5).

### Experiences of challenges of accessing healthcare

The lack of professionalism among healthcare workers results in poor accessibility to health services, which exacerbates the mental health challenges of LGBTI+ individuals. Staff attitudes that include healthcare workers imposing religious views on clients have been documented to lead LGBTI+ individuals to poor utilisation of health services (Muller 2017:6; Romani et al. 2020:240). Experiences of having to undress unnecessarily or being asked inappropriate questions aggravate the feelings of indignity among the LGBTI+ community. The current study findings are consistent with several studies that highlighted intrusive and unnecessary questioning by healthcare practitioners when dealing with some transgender individuals, which adversely leads to minority stress (McCullough et al. 2017:429; Zway & Boonzaier 2015:15). However, in contrast to the current study, psychological services in the US have been highly utilised by LGBTI+ individuals (Berke, Maples-Keller & Richards 2016:1).

### Social challenges of everyday life

Social challenges lead to the development of mental health challenges as observed from the study. As an example, the risk of public health issues is heightened by exclusion from social services. The lack of an appropriate gender marker, which is common in the study on transgender individuals, may result in poor access to services. This leads to their frustration on being questioned on their identity that results in loss of dignity and humiliation. Moreover, legal gender affirmation is linked with positive mental health in some American studies (Restar et al. 2020:6; Scheim, Perez-Brumer & Bauer 2020:e201).

In most countries, HRT is not provided to transgender individuals thus having to buy it for themselves, which facilitates psychological distress (Guethlin et al. 2021:6; Haire et al. 2021:8).

Members of the society, including healthcare professionals, are not familiar with transgender identities that place an emotional strain on transgender individuals (McCullough et al. 2017:429). Some transgender individuals experienced challenges in romantic relationship brought by issues related to transitioning and consequently feel less attractive (Mao, Haupert & Smith 2018:811; Marshall et al. 2020:373).

### Limitations

There are variety of groupings within the sexual minority with diverse challenges; for instance, only transgender individuals undergo transitioning (Hennekam & Dumazert 2023:1586). This is a serious limitation to the study, and if the study had focused on exploring challenges of a specific sub-group, rich data could have been collected.

### Recommendations

It is worthwhile to study LGBTI+ population in other geographical spaces within the country. As asserted by Stone (2018:2–3), there is need to guard against ‘metro-normativity’, which is the act of making LGBTI+ research a focus of cosmopolitan areas.

The researchers also recommend that healthcare workers be encouraged to use proper pronouns and names for clients identifying as LGBTI+ as that has been seen to nurture a safe environment for them (Kattari et al. 2020:5). In light of the several risk factors to mental health problems, there is need to develop strategies that will address them.

## Conclusion

It can be concluded that the mental health challenges for the LGBTI+ individuals are influenced by factors like experiences of stigma and discrimination, being misgendered and poor coping mechanisms to deal with the challenges. Unhealthy coping mechanisms like the problematic substance use among LGBTI+ individuals can lead to worsening of their mental health state.

The findings and literature highlight that some transgender and intersex individuals have social challenges that impact on their mental health. Transgender individuals have challenges with gender markers that are not aligned to the gender preferences. Transgender individuals’ mental health is affected by challenges with HRT accessibility as it is not readily available in government setup. They resort to buying from the private healthcare services as it is not provided for in the government setup.
